# Estimation of lung cancer risk using homology-based emphysema quantification in patients with lung nodules

**DOI:** 10.1371/journal.pone.0210720

**Published:** 2019-01-22

**Authors:** Mizuho Nishio, Takeshi Kubo, Kaori Togashi

**Affiliations:** 1 Department of Diagnostic Imaging and Nuclear Medicine, Kyoto University Graduate School of Medicine, Kyoto, Kyoto, Japan; 2 Preemptive Medicine and Lifestyle-related Disease Research Center, Kyoto University Hospital, Kyoto, Kyoto, Japan; Aix-Marseille Universite, FRANCE

## Abstract

The purpose of this study was to assess whether homology-based emphysema quantification (HEQ) is significantly associated with lung cancer risk. This retrospective study was approved by our institutional review board. We included 576 patients with lung nodules (317 men and 259 women; age, 66.8 ± 12.3 years), who were selected from a database previously generated for computer-aided diagnosis. Of these, 283 were diagnosed with lung cancer, whereas the remaining 293 showed benign lung nodules. HEQ was performed and percentage of low-attenuation lung area (LAA%) was calculated on the basis of computed tomography scans. Statistical models were constructed to estimate lung cancer risk using logistic regression; sex, age, smoking history (Brinkman index), LAA%, and HEQ were considered independent variables. The following three models were evaluated: the base model (sex, age, and smoking history); the LAA% model (the base model + LAA%); and the HEQ model (the base model + HEQ). Model performance was assessed using receiver operating characteristic analysis and the associated area under the curve (AUC). Differences in AUCs among the models were evaluated using Delong’s test. AUCs of the base, LAA%, and HEQ models were 0.585, 0.593, and 0.622, respectively. HEQ coefficient was statistically significant in the HEQ model (*P* = 0.00487), but LAA% coefficient was not significant in the LAA% model (*P* = 0.199). Delong’s test revealed significant difference in AUCs between the LAA% and HEQ models (*P* = 0.0455). In conclusion, after adjusting for age, sex, and smoking history (Brinkman index), HEQ was significantly associated with lung cancer risk.

## Introduction

Lung cancer is the leading cause of cancer-related deaths in the United States [1]. The National Lung Screening Trial has demonstrated that screening high-risk individuals using low-dose computed tomography (CT) reduced lung cancer-related mortality by 20% [2], fostering a hope that the detection of early-stage lung cancers may enable the administration of curative treatments. Moreover, of the 90 million current and former smokers in the United States, 9 million have been estimated to meet the criteria for undergoing CT screening [3]. Considering such a large number of potential screening participants, screening costs may be a major problem while determining inclusion criteria for the current screening programs.

To limit screening costs, predictive models for lung cancer risk have been investigated in previous studies [4],[5]. In addition, it has been suggested that lung cancer risk could be stratified using spirometry measurements and CT-based emphysema evaluations [3]. Several studies have examined the association between lung cancer risk and CT-based emphysema evaluation [6],[7]. A meta-analysis has confirmed significantly increased odds ratio (OR) for lung cancer when emphysema was detected through a visual assessment [7]. However, other studies have shown no such association between quantitative emphysema evaluations and lung cancer risk [6],[7],[8]. Wille et al [8] have investigated the association between lung cancer and visual or quantitative chest CT image assessments and have confirmed that neither percentage of low-attenuation lung area (LAA%) nor the 15^th^ percentile density was associated with lung cancer, although there was a significant association between lung cancer and visually assessed emphysema and interstitial abnormalities. Overall, the utility of quantitative emphysema evaluations remains controversial.

Homology methods have been used for medical image analysis in numerous studies [9–13], some of which have demonstrated that homology-based emphysema quantification (HEQ) was useful for assessing the severity of emphysema and for predicting results of visual scoring of emphysema [12],[13]; these findings suggest that HEQ may be useful for estimating lung cancer risk.

Therefore, the purpose of the present study was to evaluate whether HEQ is significantly associated with lung cancer risk and to construct and validate an estimation model for predicting lung cancer risk on the basis of HEQ and other clinical parameters. We hypothesized that HEQ is associated with lung cancer risk.

## Materials and methods

This retrospective study was approved by the institutional review board of Kyoto University Hospital (number: R1054); the requirement of acquiring informed consent was waived. We used a database of lung nodules, which was previously generated for computer-aided diagnosis [14],[15]. The database includes CT images and clinical information of 1,240 patients presenting with at least one lung nodule. The previous studies have focused on the computer-aided diagnosis system [14],[15], which directly uses characteristics related to lung nodules; thus, the purpose of the current study stands different from that of the previous studies.

### Database and inclusion criteria

A majority of the lung nodules in the database were diagnosed as one of three types: benign lung nodule, primary lung cancer, or metastatic lung cancer. In the present study, we focused on benign lung nodule and primary lung cancer. Diagnoses of all lung cancers were pathologically confirmed. The diagnosis of benign lung nodules was based mainly on their stability or shrinkage on CT scans, with the stability confirmed by a 2-year follow-up with CT; 57 of the benign nodules were pathologically confirmed. CT scans covered the entire chest and were acquired using a 320- or 64-detector row CT scanner (Aquilion ONE or Aquilion 64; Toshiba Medical Systems, Otawara, Japan), with automated exposure control. Parameters of CT scans were as follows: tube current, 109 ± 53.3 (range, 25–400) mA; gantry rotation time, 0.500 ± 0.0137 (range, 0.400–1.00) s; tube potential, 120 ± 1.69 (range, 120–135) kV; matrix size, 512 × 512; and slice thickness, 1 or 0.5 mm.

Patients who met the following three criteria were selected: (1) those with the lung nodule diagnosed as benign or primary lung cancer; (2) those whose non-contrast CT scans were available; and (3) those for whom smoking history (Brinkman Index) was clearly described.

### Emphysema quantification

The lungs were automatically segmented based on chest CT images using a dedicated algorithm [16], and three CT images of the upper, middle, and lower lung fields were selected for emphysema quantification [13],[17].

LAA% was calculated as follows. The lung area was evaluated (as a number of pixels) based on the lung segmentation of the three CT images, and the pixels within the lungs with attenuation lower than a predefined threshold were counted as low-attenuation lung pixels [18]; these values were used to calculate LAA%:

$$\text{LAA}\%=\frac{\text{Total}\;\text{number}\;\text{of}\;\text{low}-\text{attenuation}\;\text{lung}\;\text{pixels}\;\text{on}\;\text{the}\;\text{three}\;\text{CT}\;scans\;}{\text{Total}\;\text{number}\;\text{of}\;\text{lung}\;\text{pixels}\;\text{on}\;\text{the}\;\text{three}\;\text{CT}\;\text{images}}.$$

During this process, binary versions of the CT images were created, with 1 indicating a normal lung pixel or a pixel outside the lung and 0 indicating a lung pixel with attenuation below the defined threshold. These binary images were used for HEQ.

HEQ for the three CT images was performed as described elsewhere [12],[13]. The detailed process of HEQ in the present study has been described in supporting information (S1 File). The two previous studies have used the Betti numbers for HEQ [12],[13]. In a two-dimensional image, the Betti numbers of homology comprise two numbers: *b*_0_ and *b*_1_. In terms of lung CT images, *b*_0_ corresponds to the number of low-attenuation lung regions and *b*_1_ to the number of normal lung regions surrounded by the low-attenuation lung regions. On CT images, *b*_0_ and *b*_1_ are related to the holes formed by emphysema. Examples of binary images and corresponding Betti numbers are shown in supporting information (S2 File). Using dedicated software [12],[13], the Betti numbers can be calculated from the binary CT images acquired when calculating LAA%. Thresholds for both LAA% and HEQ were −950, −910, and −880 Hounsfield unit (HU).

### Statistical analysis

Differences in age, sex, smoking history (Brinkman index), malignant tumor history, lung area, LAA%, and HEQ (*b*_0_ and *b*_1_) were compared between the patients with and without lung cancer using chi-squared tests or *t*-test to investigate the association between lung cancer and these parameters. Furthermore, an estimation model for lung cancer risk was built using logistic regression. Before constructing the model, the best threshold for LAA% and HEQ was selected on the basis of results of *t*-tests. The statistical models included sex, age, smoking history (Brinkman index), LAA%, and HEQ as independent variables. The following three statistical models were evaluated: the base model (sex, age, and smoking history); the LAA% model (base model + LAA%); and the HEQ model (base model + HEQ). Model performance was assessed using the Akaike information criterion (AIC), analysis of receiver operating characteristic analysis, and the associated areas under the curves (AUCs). Difference in AUCs between the models was evaluated using Delong’s test. In addition, 10-fold cross validation was performed for the models to validate their robustness. Finally, the variable HEQ was binarized based on the empirically determined threshold, and a second HEQ model was constructed (HEQ_b_). The OR of the HEQ_b_ model was calculated to interpret the association between HEQ and lung cancer risk. *P*-values of <0.05 were considered significant. All analyses were performed using *R*-3.3.2 (available at http://www.r-project.org/).

## Results

**Fig 1:**
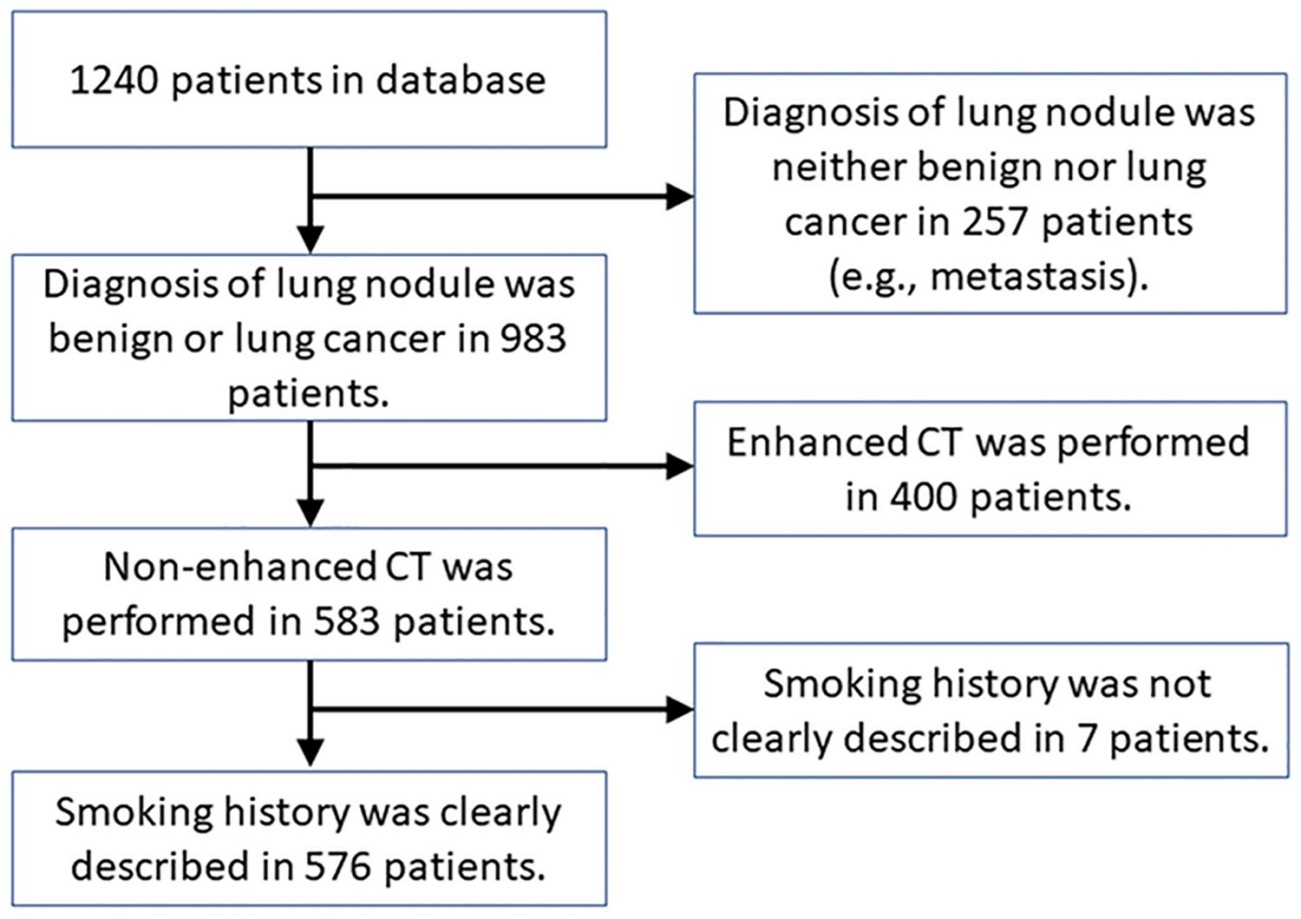
Flowchart of the patient selection process.

Fig 1 presents the patient selection process. A total of 576 patients (317 men and 259 women) were included, of which 283 were diagnosed with lung cancer and 293 with benign lung nodule. Mean (± standard deviation) patient age of 66.8 ± 12.4 years; mean Brinkman Index (representing the smoking history) was 647 ± 829.

Patient demographics and results of emphysema quantification are summarized in Table 1. Visual scores of emphysema around lung nodules were as follows: no emphysema, 419; mild emphysema, 47; severe emphysema, 110. Mean LAA% values at the three thresholds were as follows: −950 HU, 25.2% ± 10.9%; −910 HU, 41.4% ± 13.3%; and −880 HU, 53.3% ± 13.6%. Mean HEQ values at the three thresholds were as follows: −950 HU, b_0_ 7770 ± 3100, b_1_ 4930 ± 3250; −910 HU, b_0_ 3760 ± 2470, b_1_ 7300 ± 3010; and −880 HU, b_0_ 2030 ± 1850, b_1_ 7470 ± 2410.

**Table 1:** Summary of patient demographics and emphysema quantification.

Variables	All		Lung cancer		Benign lung nodule	
	Mean	SD	Mean	SD	Mean	SD
**N**	576		283		293	
**Age (years)**	66.8	12.4	68.8	9.82	64.9	14.1
**Sex (number of men)**	317		156		161	
**Smoking history (Brinkman Index)**	647	829	727	866	571	785
**Malignant tumor history**	146		67		79	
**Nodule size (mm)**	21.2	10.1	23.9	10.5	18.6	8.90
**Visual score of emphysema**						
**No emphysema**	419		199		220	
**Mild emphysema**	47		31		16	
**Severe emphysema**	110		53		57	
**Lung area of three slices (mm**^**2**^**)**	61800	11900	62300	11100	61300	12500
**LAA% at −950 HU**	25.2	10.9	25.7	10.2	24.8	11.6
**LAA% at −910 HU**	41.4	13.3	42.3	12.4	40.6	14.0
**LAA% at −880 HU**	53.3	13.6	54.4	12.7	52.3	14.3
***b***_**0**_ **at −950 HU**	7770	3100	7820	3110	7720	3100
***b***_**1**_ **at −950 HU**	4930	3250	5140	3170	4720	3320
***b***_**0**_ **at −910 HU**	3760	2470	3590	2450	3910	2480
***b***_**1**_ **at −910 HU**	7300	3010	7650	2870	6960	3100
***b***_**0**_ **at −880 HU**	2030	1850	1880	1790	2190	1900
***b***_**1**_ **at −880 HU**	7470	2410	7760	2250	7200	2520

Abbreviations: LAA%, percentage of low-attenuation lung area; *b*_0_, zero-dimensional Betti number; *b*_1_, one-dimensional Betti number; SD, standard deviation

Table 2 summarizes the results of univariate statistical analysis. Age, smoking history (Brinkman Index), b_1_ at −910 HU, b_0_ at −880 HU, and b_1_ at −880 HU significantly differed between patients with and without lung cancer. Conversely, sex, malignant tumor history, lung area, and LAA% at the three thresholds did not show statistically significant differences. Based on results in Table 2, −880 HU was selected as the best threshold for both LAA% and HEQ, and LAA% at −880 HU and b_1_ at −880 HU were used for the model construction.

**Table 2:** Results of statistical tests between patients with and without lung cancer.

Variables	*P*
Age	0.0000967
Sex	>0.999*
Smoking history (Brinkman Index)	0.0243
Malignant tumor history	0.417*
Lung area of the three slices	0.276
LAA% at −950 HU	0.312
LAA% at −910 HU	0.128
LAA% at −880 HU	0.0648
*b*_0_ at −950 HU	0.682
*b*_1_ at −950 HU	0.124
*b*_0_ at −910 HU	0.122
*b*_1_ at −910 HU	0.00536
*b*_0_ at −880 HU	0.0421
*b*_1_ at −880 HU	0.00517

*Chi-squared test. Remaining variables were tested using *t*-tests. Abbreviations: LAA%, percentage of low-attenuation lung area; *b*_0_, zero-dimensional Betti number; *b*_1_, one-dimensional Betti number.

**Fig 2:**
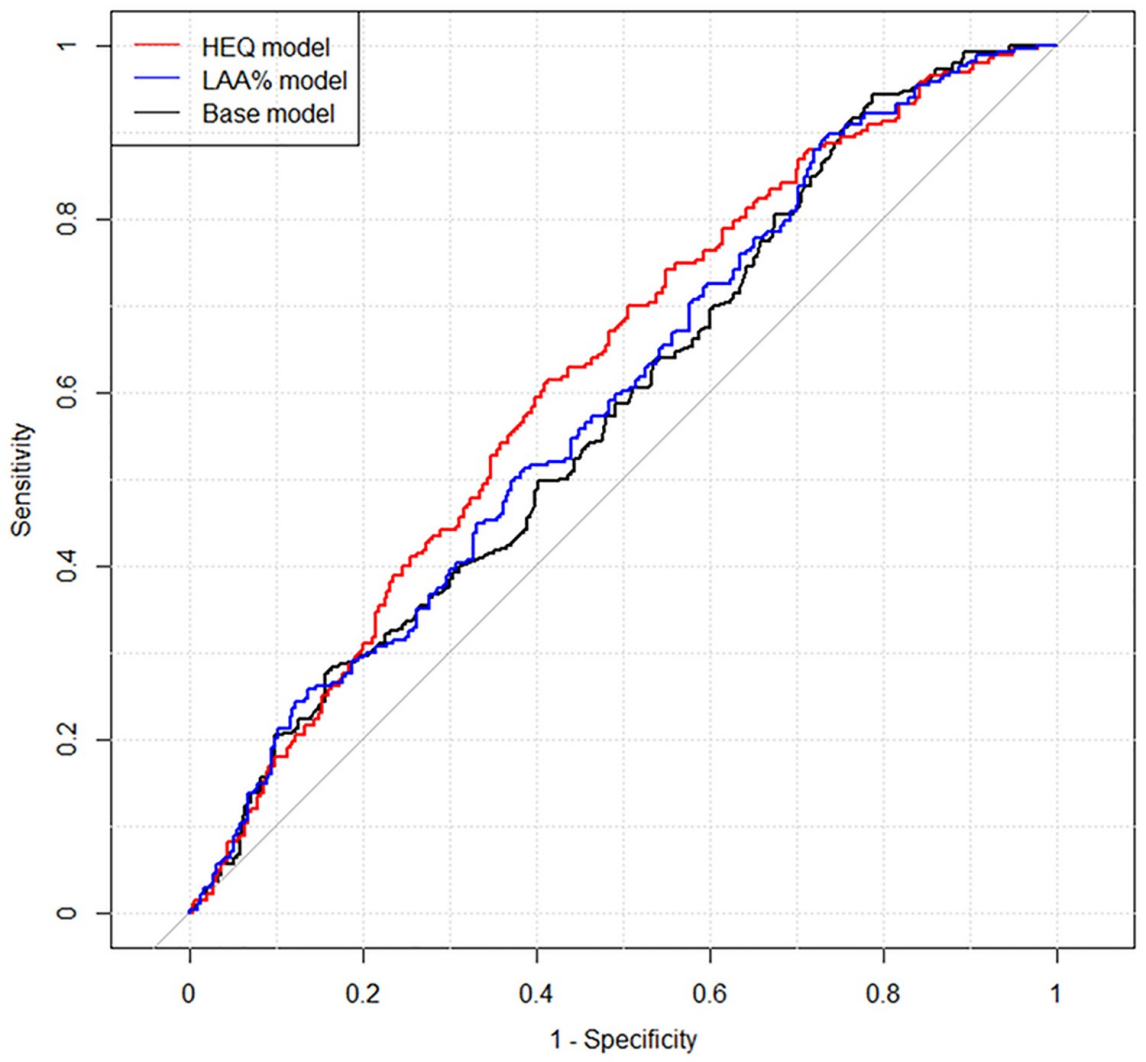
Results of receiver operating characteristic analysis for the three models for estimating lung cancer risk. The areas under the curve of the models were as follows: base model, 0.585; LAA% model, 0.593; HEQ model, 0.622. Abbreviations: LAA%, percentage of low-attenuation lung area; HEQ, homology-based emphysema quantification.

The results of the three models are summarized in Table 3. AUCs were as follows: the base model, 0.585; the LAA% model, 0.593; and the HEQ model, 0.622. Although sex was not a significant parameter in the base and LAA% models, AIC was lower when sex was included in the two models. LAA% coefficient at −880 HU was not significant in the LAA% model (*P* = 0.199). Conversely, the coefficient of b_1_ at −880 HU was statistically significant in the HEQ model (*P* = 0.00487). Delong’s test revealed significant difference in AUC between the LAA% and HEQ models (*P* = 0.0455). The receiver operating characteristic curves for the three models are shown in Fig 2.

**Table 3:** Results of the three statistical models for estimating lung cancer risk.

Model	Independent variable	Coefficient	Standard error	*P*	AIC
**Base model**	Sex = Male	−0.347	0.208	0.0959	785.9
	Age	0.0258	0.00731	0.000414	
	Smoking history (Brinkman Index)	0.000281	0.000128	0.0283	
**LAA% model**	Sex = Male	−0.379	0.210	0.0710	786.2
	Age	0.0248	0.00737	0.000758	
	Smoking history (Brinkman Index)	0.000273	0.000128	0.0332	
	LAA% at −880 HU	0.00835	0.00649	0.199	
**HEQ model**	Sex = Male	−0.438	0.212	0.0393	779.8
	Age	0.0260	0.00743	0.000479	
	Smoking history (Brinkman Index)	0.000294	0.000130	0.0238	
	*b*_1_ at −880 HU	0.000104	0.0000370	0.00487	

Abbreviations: AIC, Akaike information criterion; LAA%, percentage of low-attenuation lung area; HEQ, homology-based emphysema quantification; *b*_1_, one-dimensional Betti number

**Fig 3:**
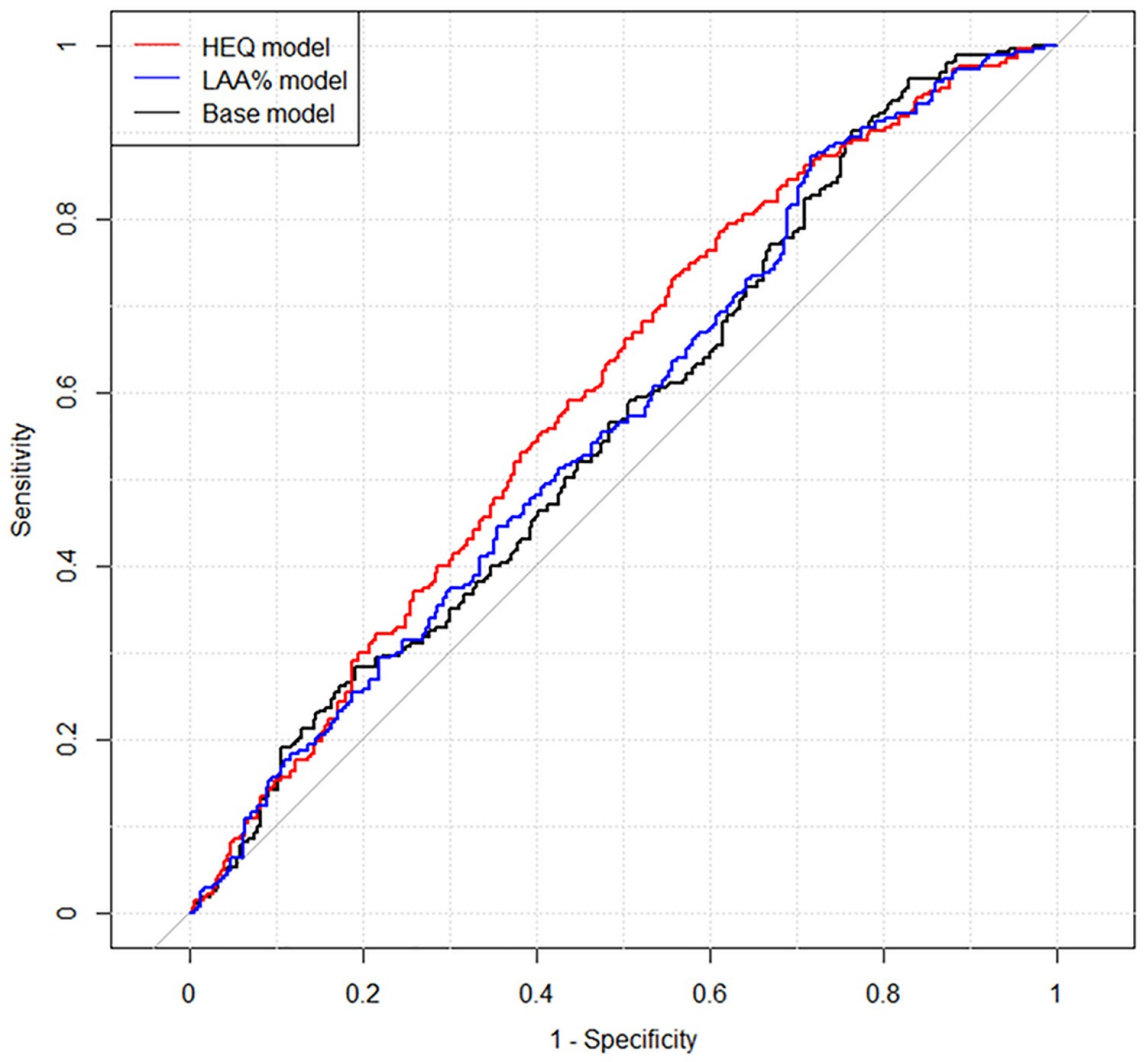
Results of receiver operating characteristic analysis for the three models with 10-fold cross validation. The areas under the curve of the models with 10-fold cross validation were as follows: base model, 0.565; LAA% model, 0.570; HEQ model, 0.602. Abbreviations: LAA%, percentage of low-attenuation lung area; HEQ, homology-based emphysema quantification.

AUCs of the models with 10-fold cross validation were as follows: base model, 0.565; LAA% model, 0.570; and HEQ model, 0.602. Delong’s test for the 10-fold cross validation revealed that the difference between the LAA% and HEQ models was significant (*P* = 0.0245). The receiver operating characteristic curves for the models with 10-fold cross validation are shown in Fig 3.

Table 4 shows the results of the HEQ_b_ model, which used binarized values for b_1_ at −880 HU. Before constructing this model, the value of b_1_ at −880 HU was replaced by 1 when it was larger than 5100 or by 0 otherwise. The AUC for the HEQ_b_ model was 0.622 without 10-fold cross validation and 0.602 with 10-fold cross validation. OR (95% confidence interval) for b_1_ as a binary variable at −880 HU was 2.28 (1.43–3.73).

**Table 4:** Results for the HEQ_b_ model using binarized values of *b*_1_ at −880 HU.

Model	Independent variable	Coefficient	*P*	OR	95% confidence interval of OR
**HEQ**_**b**_ **model**	Sex = Male	−0.387	0.0657	0.678	0.448–1.0231
	Age	0.0242	0.00114	1.0245	1.00988–1.0399
	Smoking history (Brinkman Index)	0.000298	0.0217	1.000298	1.0000478–1.000559
	*b*_1_ at −880 HU (binarized variable)	0.825	0.000725	2.283	1.426–3.725

The Akaike information criterion value and area under the curve for the HEQ_b_ model were 775.9 and 0.622, respectively. Abbreviations: HEQ, homology-based emphysema quantification; *b*_1_, one-dimensional Betti number; OR, odds ratio

## Discussion

In this study, we evaluated the association between HEQ or LAA% and lung cancer in patients with lung nodules. After adjusting for age, sex, and smoking history (Brinkman index), HEQ was significantly associated with lung cancer, while LAA% was not. Moreover, our findings indicated that the HEQ model was more effective at estimating lung cancer risk than were the base and LAA% models. In the HEQ_b_ model, OR (95% confidence interval) for the binarized b_1_ at −880 HU was 2.28 (1.43–3.73), indicating that on average, the odds of developing lung cancer in patients with high b_1_ (>5100) at −880 HU were higher by a factor of e^2.28^ ≈ 9.78 than the odds of developing lung cancer in patients with low b_1_ (≤5100).

A previous meta-analysis [7] has shown that although visually assessed emphysema using CT was independently associated with lung cancer risk, automated emphysema detection (including LAA%) was not; the pooled ORs (95% confidence intervals) were as follows: visual assessment of emphysema, 3.50 (2.71–4.51) and automated emphysema detection, 1.16 (0.48–2.81). Gietema et al have shown that for moderate-to-severe emphysema visualized on CT, the visual assessment tended to overestimate the extent of emphysema compared with LAA% at −950 HU [19]. Conversely, for smaller amounts of emphysema, the radiologists tended to underestimate the extent of emphysema compared with LAA% at −950 HU. Wilson et al have suggested that automated densitometry for emphysema evaluation was rather sensitive to distinguish clinically meaningful emphysema with respect to lung cancer risk [20]. On the basis of these results, we hypothesized that emphysema quantification should strongly correlate with visual assessment when emphysema quantification is used for the estimation of lung cancer risk.

A previous study has investigated the association of emphysema assessed by LAA% and HEQ with the visual assessment of emphysema [13] and has shown that LAA% at −875 HU and HEQ at −875 HU were strongly associated with the visual assessment values. Therefore, we used the threshold of −880 HU for emphysema quantification in the present study in addition to −950 and −910 HU. This threshold (−880 HU) is not the one typically used for LAA% in the literature, and it is difficult to compare our results of LAA% with the results of other studies.

Another previous study has shown that HEQ was useful for evaluating the spatial distribution of low-attenuation lung regions in patients with and without chronic obstructive pulmonary disease [12]. Gietema et al have shown that the visual assessment of emphysema was affected by both LAA% and the spatial distribution of low-attenuation lung regions [19]; therefore, we speculated that HEQ could be more useful for the estimation of lung cancer risk than LAA%. Our results validated this speculation, showing that b_1_ at −880 HU was significantly associated with lung cancer risk in the HEQ and HEQ_b_ models.

Similar to the previous meta-analysis [7], our results showed no statistically significant difference in the association between lung cancer risk and LAA% at the three thresholds. However, the association between LAA% and lung cancer risk improved when a relatively high threshold (−880 HU) was set. We speculated that LAA% using a higher threshold might be significantly associated with lung cancer risk. However, we did not pursue this speculation in the present study, which was designed to evaluate the association between HEQ and lung cancer risk.

This study had several limitations. First, our results were obtained retrospectively using a CT database of patients with lung nodules, who visited a single hospital [14],[15]. The patient demographics and prevalence of lung cancer in the database were different from those of patients undergoing CT screening. Frequencies of lung nodules and lung cancers in the present study were evidently different from those in CT screening; in our study, the frequencies of clinically meaningful lung nodules and lung cancers were quite high, and lung cancer prevalence was 49.1%. Therefore, our results must be evaluated in another screening population. Second, the database did not include low-dose CT. Although automated exposure control was used for scanning in our database, the radiation exposure was higher than that in CT screening. Previous studies have investigated effects of low-dose CT and iterative reconstruction on emphysema quantification, particularly on the size distribution of low-attenuation lung regions. Therefore, with iterative reconstruction, acceptable agreement in emphysema quantification between low-dose CT and standard-dose CT could be obtained [21]. Hence, we expect that the results of the present study can be replicated when low-dose CT images are reconstructed with iterative reconstruction. Third, adjustments for cofounders were limited in the present study. Only patient age, sex, and smoking history (Brinkman Index) were adjusted in the models. Previous studies have shown that the results of pulmonary function tests and clinical diagnosis of chronic obstructive pulmonary disease were significantly associated with lung cancer risk [6],[22]. In addition, several risk prediction models were investigated in previous studies [4],[5]. In a future study, we aim to explore the added value of HEQ for estimating lung cancer risk when used in combination with these cofounders and models. Finally, nodule features (such as shape and size) were not evaluated since we focused on the usefulness of emphysema quantification for estimating lung cancer risk. Combined use of HEQ and nodule features may lead to a better model for estimating lung cancer risk although such a model can only be used when lung nodules are detected.

## Conclusions

After adjusting for age, sex, and smoking history (Brinkman index), HEQ was significantly associated with lung cancer risk, and HEQ can potentially allow the stratification of lung cancer risk.

## Supporting information

S1 File Process of calculating Betti numbers of CT image. (DOCX)

S2 File Examples of binary image and its Betti numbers. (DOCX)

S3 File Raw data for Table 1. (ZIP)
