# Supplementary material for: Estimation of lung cancer risk using homology-based emphysema quantification in patients with lung nodules
Source: PLoS One. 2019 Jan 22;14(1):e0210720. doi: 10.1371/journal.pone.0210720 (PMC6342309; doi:10.1371/journal.pone.0210720)
Supplement: S1 File — (DOCX) [file pone.0210720.s001.docx]

**S1 File**

Process of calculating Betti numbers of CT image.

To perform homology-based emphysema quantification (HEQ), Betti numbers are calculated (b_0_ and b_1_) for 2D CT image. The Betti numbers are calculated by use of binarized image of CT in which each pixel can have two values: 0 and 1. To obtain binarized image of CT, CT image after lung segmentation and thresholding were used in the current study. The detail of calculating Betti numbers is described in the following sections.

1)

CT images of the upper, middle, and lower lung field were selected. The following Figure shows CT image of the upper lung field of a 76-year-old man with emphysema.

**
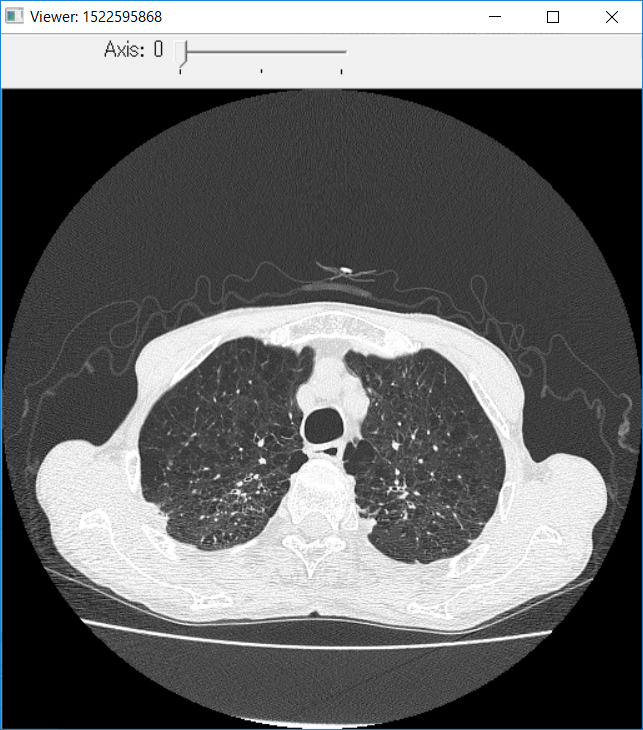
**

2)

The lungs were automatically segmented from the CT images using a dedicated algorithm^1^. The following Figure shows result of lung segmentation as binarized image, where 0 (black) indicates non-lung pixel and 1 (white) indicates lung pixel.


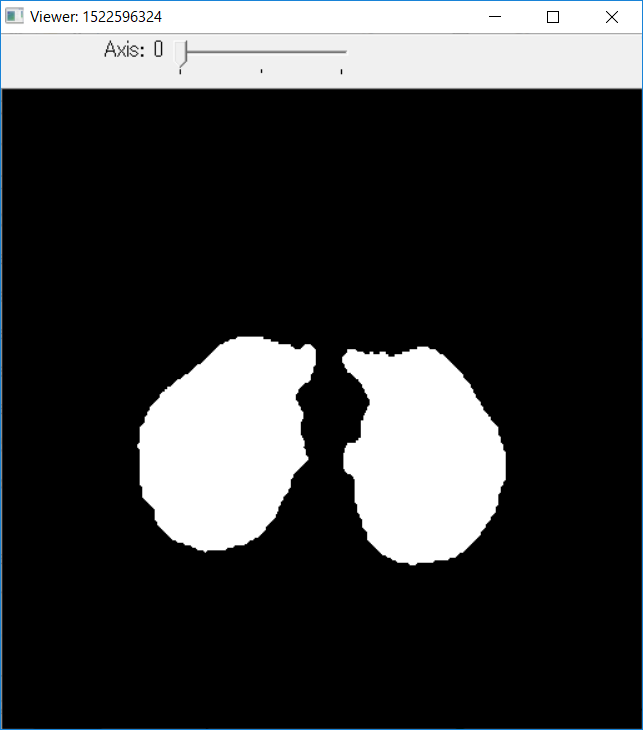


3)

Using the segmentation result, the CT image was modified, where CT density of non-lung pixel was converted to 1500 Hounsfiled unit (HU). The following Figure shows result of this modification.

**
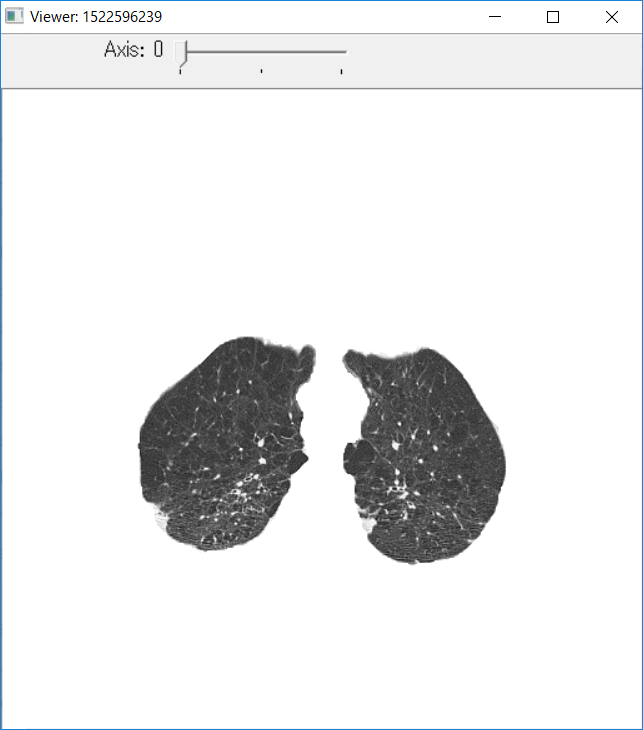
**

4)

After the process 3), CT density of each pixel in the CT image was converted to 0 or 1 by thresholding. This process produced the binarized image of CT, where 1 in the binarized image indicated a normal lung pixel or a non-lung pixel, and 0 indicated low-attenuation lung pixel. The following Figure shows result of binarization using −950 HU as threshold.
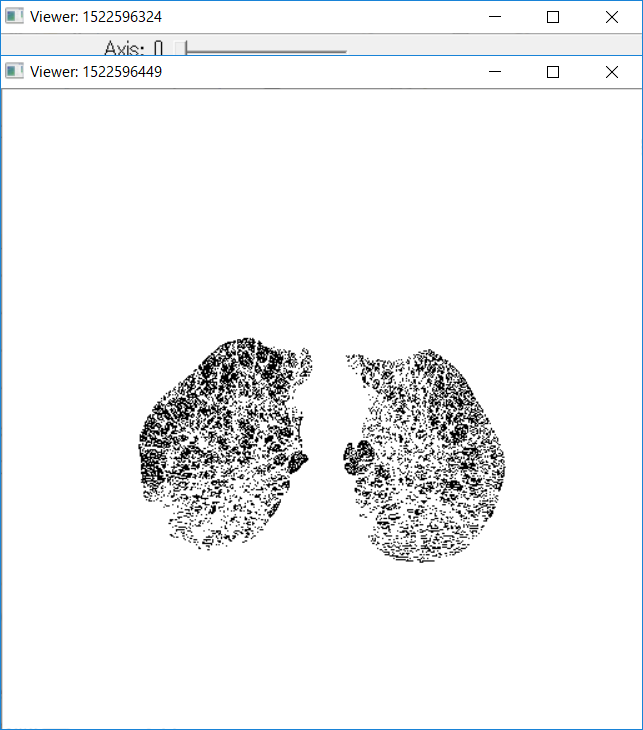


5)

The binarized image of CT was evaluated with dedicated software of homology to calculate Betti numbers. Toy examples of calculating Betti numbers are available in supplemental material2.

**References**

1. Nakagomi K, Shimizu A, Kobatake H, Yakami M, Fujimoto K, Togashi K. Multi-shape graph cuts with neighbor prior constraints and its application to lung segmentation from a chest CT volume. *Med Image Anal*. 2013;17(1):62-77. doi:10.1016/j.media.2012.08.002.
