# Supplementary material for: Estimation of lung cancer risk using homology-based emphysema quantification in patients with lung nodules
Source: PLoS One. 2019 Jan 22;14(1):e0210720. doi: 10.1371/journal.pone.0210720 (PMC6342309; doi:10.1371/journal.pone.0210720)

**S2 File**

Examples of binary image and its Betti numbers.

Note: Betti numbers (*b*_0_ and *b*_1_) of binarized image were calculated. In the images, *b*_0_ corresponds to the number of black regions; *b*_1_ corresponds to the number of white regions.

*b*_0_ = 2, *b*_1_ = 0


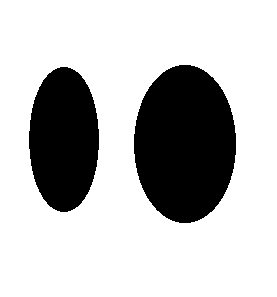


*b*_0_ = 3, *b*_1_ = 1


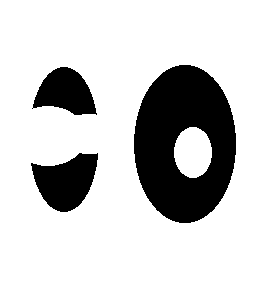


*b*_0_ = 3, *b*_1_ = 0


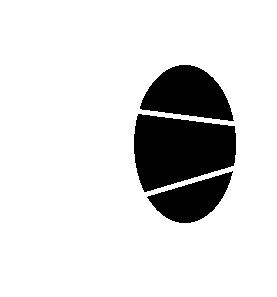


*b*_0_ = 4, *b*_1_ = 1


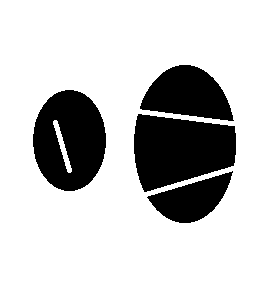

Supplement: S2 File — (DOCX) [file pone.0210720.s002.docx]
